# *Rhodococcus equi *venous catheter infection: a case report and review of the literature

**DOI:** 10.1186/1752-1947-5-358

**Published:** 2011-08-09

**Authors:** Rosalinda Guerrero, Ashish Bhargava, Zeina Nahleh

**Affiliations:** 1 Department of Internal Medicine, TTUHSC-Paul L Foster School of Medicine, 4800 Alberta Avenue, El Paso, TX 79905, USA; 2 Department of Internal Medicine, Wayne State University/Detroit Medical Center, 4100 John R, 4HWCRC, Detroit, MI 48201, USA

## Abstract

**Introduction:**

*Rhodococcus equi *is an animal pathogen that was initially isolated from horses and is being increasingly reported as a cause of infection in humans with impaired cellular immunity. However, this pathogen is underestimated as a challenging antagonist and is frequently considered to be a mere contaminant despite the potential for life-threatening infections. Most case reports have occurred in immunocompromised patients who have received organ transplants (for example kidney, heart, bone marrow) or those with human immunodeficiency virus infection. Infections often manifest as pulmonary involvement or soft tissue abscesses. Bacteremia related to *R. equi *infections of tunneled central venous catheters has rarely been described.

**Case presentation:**

We report the case of a 63-year-old non-transplant recipient, non-HIV infected Caucasian woman with endometrial carcinoma who developed recurrent bloodstream infections and septic shock due to *R. equi *and ultimately required the removal of her port catheter, a subcutaneous implantable central venous catheter. We also review the medical literature related to human infections with *R. equi*.

**Conclusion:**

*R. equi *should be considered a serious pathogen, not a contaminant, particularly in an immunocompromised patient who presents with a central venous catheter-related bloodstream infection. Counseling patients with central venous catheters who participate in activities involving exposure to domesticated animals is recommended.

## Introduction

*Rhodococcus equi *is an intracellular aerobic, Gram-positive, weakly acid-fast coccobacillus. It has been recognized as an animal pathogen since its original isolation from foals with pneumonia in Sweden in 1923 [1]. Human infection with *R. equi *is rare but is increasingly encountered in patients with human immunodeficiency virus (HIV) infection [2,3], and in solid organ transplant recipients [1,4,5]. In most of these cases, there has been a history of contact with farm animals, contaminated soil or manure, in which this organism is commonly found [2]. More than 80% of cases reported in the English medical literature have pulmonary involvement [5]. The remaining cases involve extrapulmonary sites such as soft tissues, eyes and bone [5-7]. Unfortunately, this pathogen is still underestimated as a formidable adversary in vulnerable patient populations and may be discounted by physicians and microbiology laboratories as a contaminant [2,5].

Central venous catheter-related bacteremia due to *R. equi *has been rarely described in the literature and very few cases have been reported in cancer patients [8-10]. Many cancer patients have a special form of central venous catheter known as a port. It consists of a tunneled subcutaneous reservoir with a catheter that connects to a vein (usually the subclavian or the superior vena cava).

We report the case of a 63-year-old woman without a history of organ transplantation or HIV infection, diagnosed with recurrent endometrial carcinoma and mucinous carcinoma of her appendix. During the course of her treatment, she developed recurrent central venous catheter port line infections and septic shock due to *R. equi*, which was cultured from her central venous catheter tip. She was ultimately successfully treated with removal of the catheter and a combination of antibiotics.

## Case Presentation

Our patient was a 63-year-old Caucasian woman. She does not smoke cigarettes or drink alcohol. She measures 162.5 cm in height and weighs 66 kilograms. She had no significant family history. She was initially diagnosed with stage IIIA endometrial carcinoma for which she underwent a hysterectomy and bilateral salpingo ophorectomy followed by pelvic external beam radiation therapy. Two years later, she developed recurrent disease with widespread peritoneal carcinomatosis. An incidental mucinous carcinoma of the appendix was also found during surgical exploration. A central venous catheter port was placed and she received chemotherapy with 5-fluorouracil, leucovorin, and later, irinotecan, capecitabine and oxaliplatin. Her disease progressed despite multiple lines of chemotherapy. During the course of her cancer treatment, our patient also developed a series of complications which included tumor-related right ureteral obstruction requiring a nephrostomy placement, a sigmoid-vaginal fistula necessitating a colostomy with reversion of the ureteral stent, small bowel obstruction and ileostomy placement, short bowel syndrome and significant weight loss requiring total parenteral nutrition. Our patient recovered gradually and was able to receive subsequent courses of chemotherapy consisting of capecitabine, an oral chemotherapy agent classified as an antimetabolite, and oxaliplatin, an intravenous platinum-based chemotherapy agent classified as an alkylating agent. She developed predictable chemotherapy-related adverse events including diarrhea and hand-foot syndrome, but was able to continue her treatment intermittently. She started developing fever and tenderness at the site of her central venous catheter. Blood cultures were repeatedly negative. Our patient had not received antibiotics within the eight weeks prior to this event. She was not neutropenic. Laboratory results reflected a white blood cell (WBC) count of 12,100 cells/μL, with 87% neutrophils. Urine and stool examination revealed no abnormal findings. Liver and renal function tests and a chest X-ray were normal. Blood cultures taken from both the central venous catheter and peripheral vein were reported to have no growth after 96 hours incubation. She received empiric vancomycin for two weeks and all signs of infection, including fever, resolved with normalization of WBC count to 7500 cells/μL and 67% neutrophils. However, four weeks later, she was admitted to the intensive care unit with a septic shock picture; she had fever, hypotension and tenderness at the site of the central venous catheter, which was then removed. WBC count at this time was 18,400 cells/μL with 93% neutrophils. A chest X-ray was negative for pulmonary disease and sputum cultures revealed no microbial growth. She was empirically treated with intravenous ticarcillin/clavulanate, ciprofloxacin and vancomycin. Cultures from the catheter tip as well as blood cultures were submitted for analysis. Two days later, large, irregular mucoid colonies grew and were non-fermentative, distinguishing them from diphtheria colonies. Further incubation grew characteristic salmon-colored colonies of *R. equi*. Microbiologic characteristics of the pathogen were as follows: catalase and urease positive; oxidase, carbohydrate fermentation, mannitol, indole, and citric acid negative; *equi *factors positive. The cultured organism was found to be resistant *in vitro *to penicillins and susceptible to erythromycin, gentamicin, tobramycin, vancomycin, imipenem/cilastatin and rifampin. Our patient's antibiotic regimen was modified to include the combination of a β-lactam antibiotic (imipenem/cilastatin) and a bactericidal antibiotic rifampin. She received imipenem 500 mg intravenously every 8 hrs for 14 days and rifampin 600 mg orally daily. She was discharged on oral rifampin after resolution of her symptoms and two negative blood cultures were obtained. She continued rifampin for twenty-one days and made a full recovery.

## Discussion

The first human case with *R. equi *infection was reported in 1967 in a patient with autoimmune hepatitis who was undergoing treatment with prednisone and 6- mercaptopurine [11]. He worked in a stockyard cleaning animal pens and subsequently developed lung and subcutaneous abscesses. Since then, human cases of *R. equi *infection have been described in immunocompromised patients. In this subpopulation of patients, such infections portend high mortality rates and usually require prolonged treatment with multiple antibiotics [2-8,12]. In contrast, immunocompetent patients respond well to shorter courses of antibiotics, usually with a single agent [13,14]. With the exception of Antarctica, *R. equi *has been identified in soils all over the world, in fresh and sea water and in animals including horses, cattle and wild birds [5]. Human infection can be acquired through inhalation from the soil, inoculation into a wound or ingestion and passage through the alimentary tract [4,5]. Other routes of acquisition include nosocomial spread, human colonization and person-to-person transmission [15]. Exposure to domesticated animals such as horses and pigs has been reported in some cases of infection [16]. Our patient may have potentially acquired *R. equi *via exposure to contaminated horse fibers that she uses for her sculptures.

In immunocompromised patients, pulmonary involvement is common, with necrotizing pneumonia being the most frequent presentation [4]. Infection with this organism can be life-threatening and the required treatment is often lengthy. In immunocompetent patients, pulmonary infections are also common and account for 42% of reported cases [14]. Pulmonary infections have a relapsing and remitting course with intermittent bacteremia. The onset of symptoms is usually insidious and may occur over a span of days to weeks, with the patient presenting with fever, non-productive cough, dyspnea and pleuritic chest pain. In some cases, weight loss and hemoptysis, severe enough as to require blood transfusions, have also been noted. Chest radiographs typically reveal pulmonary infiltrates with single or multiple lung segments, mainly in the upper lobes. The radiographic changes bear a striking resemblance to those changes that occur with fungal or tubercular infections. Cavitations, pleural effusions or empyema evolve over a two to four week period [4]. Primary extrapulmonary manifestations are unusual and occur for the most part secondary to hematogenous dissemination. Examples include subcutaneous nodules, brain and renal abscess, lymphadenitis, endophthalmitis and osteomyelitis [17].

Blood cultures are positive in more than one-half of immunocompromised patients with *R. equi *infection compared to only 10% of normal hosts [18]. Cultures of *R. equi *grow easily under aerobic conditions on non-selective media. Large, irregular, highly mucoid colonies usually grow optimally at 30°C and turn to a salmon-pink color within 48 hours [18]. Further incubation leads to release of its red pigment leading to its characteristic salmon-colored colonies. It is non-fermentive which differentiates *Rhodococcus *from *Corynebacterium*. *R. equi *is catalase- and urease-positive, and oxidase-negative. Biochemical kits are now available which facilitate identification of *R. equi*. Our patient had recurrent infections of the central venous catheter which eventually led to its removal. The first set of blood cultures was reported as negative. This may have occurred, in part, due to the fact that infections with *R. equi *are missed because of incomplete or improper identification of the organism [4,5]. In addition, the appearance of *R. equi *as a Gram-positive, weakly acid-fast, diphtheroid-like organism may lead to mistaken identity with a component of the normal flora or a contaminant (a diphtheroid, a micrococcus, or a *Bacillus *species) [2]. Therefore, a high degree of suspicion should be exercised in susceptible patients. Once *R. equi *is cultured from a sterile site, it should never be considered a contaminating diphtheroid. Septic shock in our patient resulted in part from recurrent infections of the central venous catheter.

Infections with *R. equi *may be life-threatening due to the toxicity of this organism, which is mediated by the presence of large plasmids that encode proteins necessary for virulence inside the cell [19]. This coding process ensures the pathogen's ability to persist and destroy macrophages in the immunocompromised patient [20,21]. Infection of macrophages with *R. equi *results in cytotoxicity, particularly in high bacterial loads [22], and is regulated by virulence-associated plasmids (VAP) [22,23]. More recent work suggests that the type of plasmid that is overcome by a specific *R. equi *strain determines its host specificity, as described by the plasmid-typing scheme known as TRAVAP [23]. TRAVAP is an acronym that represents a polymerase chain reaction (PCR) typing system for *R. equi *in which three plasmid gene markers are evaluated. The first marker is the traA which is found in the conserved conjugal transfer machinery while vapA and vapB are found in two different plasmid subpopulations [23]. In humans and in healthy or afflicted animals with tuberculous-appearing lesions, isolated strains of *R. equi *often possess a VAP coding for a surface-localized 20-kDa protein, and has been referred to as " vapB" due to its high homology to the vapA protein [19,24]. VapA and vapB sequences are strongly related to each other (83.6% identity) [24] and so are the plasmids encoding them [25]. Possession of certain vaps seems to be specific for strains infecting foals, pigs or cattle [19], but it is likely that *R. equi *infections of humans are not determined by particular plasmids but by the basal and chromosomally determined pathogenic potential of *R. equi *[26]. Chromosomally encoded factors involved in *R. equi *virulence have been reported [26,27] The mortality rate for *R. equi *infection among immunocompetent patients is approximately 10%, compared with rates of 20-55% among immunocompromised patients, in particular, those with HIV [7]. In humans, *R. equi *typically resides in, and destroys, macrophages, making it difficult to eradicate especially in immunocompromised patients [12]. This is exemplified in our case. The organism persisted and colonized the central venous catheter after vancomycin therapy, despite therapeutic trough levels, with the organism remaining susceptible to vancomycin. Another factor that may have led to therapeutic failure is the fact that this organism can inhibit macrophage phagosome-lysosome fusion and survives within the cell. Therefore, vancomycin monotherapy may not be the ideal approach to the management of central venous catheter bacteremia with *R. equi*, despite its susceptibility to vancomycin. Combination antimicrobial therapy using bactericidal and intracellular-active agents should be considered. Also, prompt removal of the infected central venous catheter is necessary for adequate infection control, as was the case in our patient.

Therapeutic failure may also occur following a deficient course of treatment. Based on similar experiences with difficult-to-treat organisms like *Mycobacterium tuberculosis*, and on the fact that distant relapses of rhodococcus infection are common, prolonged therapy is recommended [28]. Although there is no consensus on the optimal duration or regimen of antibiotic treatment, the use of combination therapy may decrease the risk of developing resistance during therapy, which has been described with penicillin and other β-lactam antibiotics. A carbapenem and a glycopeptide, such as meropenem and vancomycin, are good choices [29]. The combination of macrolides and rifampin can also be considered [30]. Other combinations may include a macrolide antibiotic such as erythromycin along with rifampin, vancomycin, fluoroquinolones, aminoglycosides or broad spectrum β-lactam antibiotics such as imipenem/cilastatin [28-32]. After initial improvement, the patient can be treated with an oral regimen that could include combinations of quinolones, tetracycline, macrolides, and rifampin.

The optimal duration of treatment is unknown. Our patient was successfully treated with a combination of imipenem/cilastatin and rifampin for 14 days, and then continued oral rifampin for 21 days. Due to the intracellular nature of the pathogen, which concentrates in granulocytes and macrophages [31], a prolonged treatment course is advised in immunocompromised patients due to frequent relapses following abbreviated treatment courses. Monotherapy with penicillin and most other β-lactam antibiotics should be avoided even if *R. equi *is initially sensitive, since β-lactam resistance may develop during therapy [2]. Also, the minimal inhibitory concentrations of rifampin and erythromycin for *R. equi *strains isolated within the last 10 years have been rising [33] and the emergence of resistant strains to different antibiotics have been reported [34]. These findings highlight the need for strategies other than antibiotic therapy to prevent or treat *R. equi *infections, such as applications of gallium nitrate and vaccination methods [35-41]. In the case of patients taking immunosuppressive therapy following organ transplants, the challenge in the treatment of *R. equi *is the possible interactions of common combination therapy like macrolide antibiotic and rifampin with immunosuppressive agents like tacrolimus or cyclosporine, which are routinely used in these patients. In one case report of a heart transplant patient on immunosuppressive therapy, the excellent response to treatment of *R. equi *occurred following the treatment with a combination of minocycline and a fluoroquinolone [42]. Overall there is no agreement on the treatment of *R. equi *infections in transplant recipients receiving immunosuppressive therapy. A review of the literature and scattered case reports describes different combinations that may prove beneficial in organ transplant recipients, such as a case of a kidney transplant patient who had good results using the combination of carbapenem and teicoplanin [43]. Synergistic combinations of medications are also key, as was demonstrated in human isolates determined by fractional inhibitory concentration indices. Such combinations included: rifampin-minocycline, erythromycin-minocycline, rifampin-erythromycin and imipenem-amikacin [44]. Weinstock and Brown [45] proposed an algorithm for the management of *R. equi *infections in immunocompromised hosts. They recommended an initial treatment with two agents to avoid development of resistance. Vancomycin, carbapenems, quinolones, erythromycin, and rifampin are reasonable first choices. Careful scrutiny of all other medications is mandatory if drug interactions are to be avoided. Later adjustment of therapy based on susceptibility data is recommended. After two weeks of intravenous therapy and attendant clinical improvement, oral antibiotics may be substituted with rifampin, erythromycin, or ciprofloxacin [45]. Six months or more of therapy may be required for lung, bone and joint, and cerebral infections [45]. Novel diagnostic techniques using specific quantitative PCR should be further explored [46,47].

## Conclusion

Human infection with *R. equi *should be considered when evaluating immunocompromised patients with a central venous catheter and fever in the setting of exposure to farm animals. Microbiologists should be familiar with the growth requirements and biochemical properties of this organism. If the pathogen is suspected and identified, the tunneled central venous catheter should be removed to prevent recurrence. Due to the fact that virulent strains of *R. equi *are resistant to phagocytosis and intracellular killing by macrophages, patients should receive a combination of bactericidal and intracellular-active agents that will penetrate cells, such as rifampin and macrolide antibiotics. A prolonged course of an oral antibiotic following initial intravenous therapy has been suggested in the light of frequent relapses with shorter courses. Counseling should be provided to immunocompromised patients inclined to participate in activities involving exposure to domesticated animals or their products.

## Consent

Written informed consent was obtained from the patient for publication of this case report. A copy of the written consent is available for review by the Editor-in-Chief of this journal.

## Competing interests

The authors declare that they have no competing interests.

## Authors' contributions

ZN developed the manuscript idea, gathered case details, performed the history, physical exam and the treatment of the patient, and co-wrote and edited the manuscript. RG was a major contributor in writing the manuscript. AB reviewed the literature and contributed to writing the manuscript. All authors read and approved the final manuscript.
